# Characterization of a Hydrogel Composite Containing Bioactive *Moringa* as a Novel Pulp-Capping Material

**DOI:** 10.3390/polym17192626

**Published:** 2025-09-28

**Authors:** Mustafa Tariq Mutar, Anas F. Mahdee

**Affiliations:** Restorative and Aesthetic Dentistry Department, College of Dentistry, University of Baghdad, Baghdad 10047, Iraq; mostafa.abd2404p@codental.uobaghdad.edu.iq

**Keywords:** hydrogels, polyvinyl alcohol, hyaluronic acid, sodium alginate, *Moringa oleifera*, dental pulp capping

## Abstract

Hydrogels are hydrophilic biocompatible polymers that can be used as a drug delivery material in different medical branches, including vital pulp therapy. The aim of this study is to characterize the physical and biological properties of the newly developed formula as a candidate direct pulp-capping material. The hydrogel composite was prepared from natural and synthetic origins (polyvinyl alcohol (PVA), hyaluronic acid (HA), and sodium alginate (SA)) with the incorporation of bioactive *Moringa*. Different formulas of hydrogel containing different concentrations were evaluated for physicochemical (FTIR, XRD, SEM, degradation, and swelling), mechanical (viscosity, folding endurance, film thickness), and biological (antioxidant, antibacterial, and cytotoxicity) properties. FTIR and XRD confirmed successful incorporation and partial cross-linking between *moringa* and hydrogel compounds. At low concentrations of *moringa*, the hydrogel formula showed integrity, scavenging activity, and homogeneity. The *moringa*-loaded films showed concentration-dependent antioxidant and antibacterial properties, especially at higher concentrations, with acceptable cytocompatibility. The low concentration of *moringa* (0.5%) may be considered a promising candidate as a novel pulp-capping agent supporting tissue healing and regeneration.

## 1. Introduction

The dentine–pulp complex shows the capacity to regenerate when a favorable environment is available [1]. Among these is the use of pulp-capping agents: medical materials which can provide a suitable bioactive, antimicrobial, and sealing environment for the pulp to heal [2,3]. These materials, such as calcium hydroxide and calcium silicate-based cements, can induce pulp–dentine complex repair [4,5]. However, the induced tissue is still far from the original and commonly associated with tunnel defects, superficial necrosis, chronic inflammation, and intrapulpal calcification [6]. These shortcomings could be related to the type of these capping materials, which are mainly cements, rather than other more biological formulations.

Various experimental materials containing herbal extracts or products were suggested for pulp regeneration. *Moringa oleifera* leaf extract is one of these, which has shown several advantages, including its antibacterial, biocompatible, immunomodulatory, and bioactive properties [7,8,9]. Both ethanolic and aqueous extracts have been reported to induce alkaline phosphatase levels in human cultured pulp stem cells, leading to the formation of calcified nodules similar to MTA [10]. However, these extracts still need a suitable carrier to hold them actively above the exposed pulp region.

Hydrogels are hydrophilic biocompatible polymers that can be used as a drug delivery material in different medical branches, including vital pulp therapy [11,12]. They can absorb water, expand, and release their loaded molecules [12]. Among these is polyvinyl alcohol (PVA), which is a synthetic biocompatible polymer that can produce a thin adhesive film when it dries [13]. It has been used as a luting material for attaching bioactive materials to the surfaces of other hydrophobic mesh scaffolds coated with poly lactic acid, which are suggested for pulp regeneration [14,15]. In addition, PVA has several medical applications, such as wound dressing, drug delivery, contact lenses, and the lining of artificial hearts [13]. However, it has several drawbacks, such as lack of flexibility and low hydrophilicity [16]. To overcome these limitations, a combination with other hydrogels (such as hyaluronic acid (HA) and sodium alginate (SA)) has been introduced as a composite type for different medical applications, such as wound dressing and cartilage replacement [17,18]. This combination has been reported to have better hydrophilicity, drug delivery, flexibility, cell adhesion, and biocompatibility [17]. Therefore, incorporation of *Morina* leaf extract within a hydrogel composite can be introduced as an alternative pulp-capping material. Thus, this study aims to fabricate and characterize the physical and biological properties of this newly developed formula as a candidate for direct pulp-capping materials.

## 2. Materials and Methods

### 2.1. Plant Extract Preparation

*Moringa oleifera* plant leaves were collected from a local agricultural nursery and identified by a plant identification expert. Leaves were left in the shade at room temperature to achieve desiccation and then ground by an electrical blender to produce dried leaf powder. Then, ethanolic extract was prepared by the cold maceration method as described by Shafiq and Mahdee [10], by adding leaf powder into a beaker containing 80% ethanol (RCI Labscan Limited, Bangkok, Thailand) in a proportion of 1:10; mixing for 3 h by a magnetic stirrer (JOANLAB Equipment, Ningbo, China), and then storing for 72 h at room temperature. The mixture was filtered by Whatman filter paper no. 1 (W&R Balston Limited, Maidstone, England) before being concentrated by a rotary evaporator (BUCHI, Flawil, Switzerland) at 45 °C.

### 2.2. High-Performance Liquid Chromatography (HPLC)

The test was conducted utilizing HPLC (Shimadzu, Kyoto, Japan), with the separation executed on the ODS C18 column (4.6 × 150 mm^2^) with a particle size of 5 µm. The mobile phase comprises a mixture of 0.01 M phosphoric acid (mobile phase A) and acetonitrile (mobile phase B), at a temperature of 40 °C, injection concentration of 10 µg/mL, injection volume of 20 µL, flow rate of 0.6 mL/min, and the wavelength of 254 nm for detecting phenolic compounds.

The following standards of known concentration (10 ppm) were employed: quercetin, kaempferol, rutin, myricetin, apigenin, isorhamnetin, gallic acid, chlorogenic acid, and vanillic acid (HyperChem, Hangzhou, China). For unidentified samples, 10 mg of the dried extract was dissolved in an acetonitrile/phosphoric acid binary solvent.

The concentration of identified compounds was calculated according to the following formula:

(1) $$concentration\;of\;unknown=\frac{area\;of\;unknown\times concentration\;of\;known}{area\;of\;known}$$

### 2.3. Hydrogel Composite

#### 2.3.1. Hydrogel Composite Preparation

This includes the preparation of PVA solution by adding fully hydrolyzed 1799 PVA powder (M_w_ 44,000 Da, HyperChem, Hangzhou, China) to deionized water at 90 °C and stirring at 700 rpm for 2 h to obtain a homogenous solution of 10% PVA (solution A).

Similarly, 0.25% hyaluronic acid (HA) and 3.75% sodium alginate (SA) were also prepared. Sodium hyaluronate with M_w_ (0.75~1.50) × 10^6^ Da (Hyper Chem, Hangzhou, China) and SA M_w_ 90,000 Da (HyperChem, Hangzhou, China) powders, respectively, were mixed separately with deionized water at 45 °C and stirred at 700 rpm for 0.5 h until homogeneous solutions B and C were obtained. Then, these three solutions (A, B, and C) were mixed in proportions of 60:20:20%, respectively, at 45 °C and stirred at 700 rpm for 0.5 h to obtain a homogenous composite mixture.

#### 2.3.2. Addition of Moringa to Hydrogel Composite

*Moringa* dried extract was weighed and added to composite polymer w/w in different concentrations (0.5%, 5%, 7.5%, 10%, and 20%) at 45 °C and 700 rpm, stirring for 0.5 h until a homogenous mixture was obtained.

The upcoming characterization tests have analyzed either a fresh hydrogel mix or film (with or without *moringa*). Films can be fabricated through the solvent casting method by pouring the mixture of 1 mL polymer into a 2 × 3 cm Teflon mold and drying it in an oven (ZX Insturment, Beijing, China) at 40 °C for 24 h.

### 2.4. pH Value Assessment

The pH meter (Hanna, Woonsocket, RI, USA) was calibrated using a standard buffer solution (pH: 7.01) by single-point calibration, and then the device was immersed in a beaker containing 5 mL of formulas (hydrogel composite, with 0.5% and 10% *moringa*) in gel state. The readings were recorded when the measures were stable.

### 2.5. Antibacterial Assay (Well Diffusion Method)

Streptococcus mutans isolated from patients attending the College of Dentistry, University of Baghdad, were analyzed by microscope and confirmed by VITEK II. The bacteria were cultured in a broth made from brain and heart infusions (HiMedia, Mumbai, India) and incubated overnight at 37 °C using an electro-thermal incubator. The bacterial suspension turbidity was adjusted with normal saline to achieve a 0.5 McFarland standard (1.5 × 10^8^ CFU/mL). Sterile Mueller–Hinton agar (Thermo Fisher Scientific, Waltham, MA, USA) was poured in 3 plates and left to solidify before spreading bacterial suspension over the plates. Four holes of 6 mm diameter were made on each plate to test 5%, 7.5%, 10%, and 20% concentrations of *moringa* hydrogels. The plates were incubated overnight at 37 °C before measuring the inhibition zones.

### 2.6. Antioxidant Assay (DPPH Test)

The antioxidant effect of hydrogel films with different concentrations w/w (0.5%, 5%, 10%, and 20%) of *moringa* was measured using the DPPH (2,2-diphenyl-1-picrylhydrazyl) scavenging method. Film extracts were obtained by dissolving films in 5 mL of phosphate buffer saline (PBS) for 1 day in a thermo-electronic incubator at 37 °C. The DPPH was dissolved in absolute methanol at a concentration of 50 ppm. Extracts were mixed with the DPPH solution inside a dark chamber before incubation for 1 h, and the concentration of the remaining material was measured using a UV–vis spectrophotometer at wavelength 517 nm (Shimadzu, Kyoto, Japan). The DPPH solution was used as a blank, and ascorbic acid was used as a negative control. The scavenging activity was measured according to the following formula:

(2) $$\%\;scavenging\;activity=\frac{{Abs}_{blank}-{Abs}_{sample}}{{Abs}_{blank}}\times 100$$

### 2.7. Cytotoxicity Test (MTT Assay)

The primary objective of this test was to identify the proper *moringa* hydrogel concentration with minimal cytotoxic effect on normal human fibroblasts (NHF). The cell line was cultured in minimum essential media (MEM) (US Biological, Salem, MA, USA) supplemented with 10% (v/v) fetal bovine serum (FBS) (Capricorn-Scientific, Ebsdorfergrund, Germany), 100 IU penicillin, and 100 µg of streptomycin (Capricorn-Scientific, Ebsdorfergrund, Germany) and incubated in a humidified environment at 37 °C until it reached a 90% confluent monolayer. The cells were counted using a hemacytometer and seeded at a concentration of 1 × 10^4^ cells in a 96-well microplate (NEST Biotech, Wuxi, China) and incubated at 37 °C for 72 h. Cytotoxicity was investigated through the 3-(4,5-dimethylthiazol-2-yl)-2,5-diphenyltetrazolium bromide (MTT) assay (Elabscience, Wuhan, China).

Hydrogel film extracts of different formulas (hydrogel composite, 0.5%, 5%, 10%, and 20% *moringa*) were prepared according to ISO standards 10993-12; a film of surface area 6 cm^2^ was immersed in 1 mL of serum-free media at 37 °C for 24 h [19]. To remove undissolved particles and ensure germ-free extract, the eluate was filtered by a Millipore syringe filter of 0.20 µm.

The wells that received no treatment were used as a control. Two plates were prepared with different incubation periods (24 h and 72 h) at a given temperature and atmosphere for this incubation (aerobic or anaerobic). Then, 28 µL of MTT dye solution (2 mg/mL) was added to each well, incubated for 3 h, then 100 µL of DMSO was added, followed by further incubation for 15 min, before measuring optical density at 492 nm by using a microplate reader. Cytotoxicity % was calculated using the following equation:

(3) $$Cytotoxicity\;\%=\frac{\left(OD\;control-OD\;sample\right)}{OD\;control}\times 100\%$$

OD control is the mean optical density of untreated wells, and OD sample is the optical density of treated wells.

Based on the results of the above tests, only 0.5% and 10% of the *moringa* hydrogel were processed for further tests.

### 2.8. Drying Time

This is a modified method mentioned by Kathe and Kathpalia (2017) to detect the drying time of film on the skin of volunteers; therefore, to simulate the clinical situation, simulated Class V cavity preparations were performed on natural human premolars teeth [20]. All formulas (hydrogel composite, with 0.5% and 10% *moringa*) were applied to the axial wall of the cavity using a micro-brush, followed by the application of gentle air for 20 s. The time during which the material lost its glossy surface and no longer adhered to a new micro-brush was quantified in seconds and considered as an estimated drying time. The samples were performed in triplicate for each formula.

### 2.9. Film Thickness

Films were made (n = 3) from three different formulas (hydrogel composite, with 0.5, and with 10% *moringa*) and measured at six random points by a stainless steel digital vernier caliper (Kales Tool, Shanghai, China). The average film thickness was calculated for each film thickness and expressed in mm.

The retraction ratio was evaluated according to the following equation:

(4) $$\mathrm{RR}=\frac{T1-T2}{T1}\times 100\%$$

RR is the retraction ratio, T1 is the thickness of poured hydrogel in the mold, and T2 is the final thickness of the film after drying.

### 2.10. Fourier Transform Infrared Spectroscopy (FTIR)

This test was performed utilizing FTIR (Shimadzu, Kyoto, Japan) within the frequency range 4000–400 cm^−1^ with a resolution of 2.0 cm^−1^ in transmission mode via the KBr pellet technique to detect the chemical structure of PVA, HA, SA, and *moringa* extract alone and as a composite mixture.

### 2.11. X-Ray Diffraction (XRD)

To evaluate the crystallinity of different formulas, hydrogel composite films with 0.5% and 10% *moringa* were prepared and tested by an X-ray diffractometer PW1730 (Philips, Eindhoven, The Netherlands) with Cu Kα radiation (λ = 1.54 Å) at a temperature of 25 °C, in the 2θ range of 8.79−79.99° with a 0.05 step every 1 sec.

### 2.12. Shear Viscosity

Three formulas (hydrogel composite, with 0.5% and 10% *moringa*) were determined by placing 2 mL of the gel for each formula on the plate of the viscometer (Brookfield, Middleboro, MA, USA). The viscosity was determined by a CP 41 spindle (cone angle = 3° and cone radius = 2.4 cm) and a shear rate of 2 N/sec at 37 °C. The viscosity was measured against the shear rate in the range of 0.02–60.00.

### 2.13. Scanning Electron Microscopy (SEM)

The film specimens (n = 3) of different formulas (hydrogel composite, with 0.5%, and with 10% *moringa*) were analyzed by Axia ChemiSEM (ThermoFisher Scientific, Waltham, MA, USA) with an acceleration voltage of 30 kV. The specimen was sputtered with a thin gold layer to avoid a buildup of electrostatic charge on the surface. The secondary electron imaging mode was used to detect surface topography by using the Everhart–Thornley Detector. The magnification was set in a range from 70× to 4000×.

### 2.14. Folding Endurance

To evaluate the mechanical performance of the hydrogels, films from different formulas (hydrogel composite, with 0.5%, and with 10% *moringa*) were bent several times at the same point until breaking or passing 300 times without breaking [21]. This procedure was performed in triplicate for each formula.

### 2.15. Degradation Rate

Different formulas of films (n = 3) of hydrogel composites without and with *moringa* (0.5% and 10%) were initially weighed by an electronic balance (JOANLAB, Ningbo, China). The samples were fully immersed in 5 mL of PBS (pH = 7.4; Chemical Point, Manchester, UK) and incubated (ZXInstrument, Beijing, China) at 37 °C. The samples were dried at different intervals (1d, 3d, and 7d) by a drying oven at 40 °C before weighing. The solubility was checked according to the following equation:

(5) $$Degredation\;\left(\%\right)=\frac{\left(Wo-Wi\right)}{Wo}\;\times \;100$$

The procedure was performed in triplicate and statistically analyzed.

### 2.16. Swelling Index

Similar films from all formulas (n = 3) were weighed, then immersed in 5 mL of PBS and incubated for 1 min, 5 min, and 10 min at 37 °C. After each time interval the films were removed, and excess water was dried by filter paper, then weighed.

The swelling index was calculated according to the following equation:

α = (W*_t_* − W*_o_*)/W*_o_* (6)

W*_t_* is the weight of the film at time t, and W*_o_* is the weight of the film at time zero.

### 2.17. In Vitro Drug Release

Hydrogel film containing *moringa* of different concentrations (0.5% and 10% w/w) was immersed in 5 mL PBS and incubated at 37 °C. The sample aliquot of 5 mL was decanted into disposable graduated test tubes at different intervals (1 d, 3 d, and 7 d) and filtered by 45 µm of Millipore filter to remove undissolved particles. A volume of 5 mL of fresh PBS was added to substitute the measured aliquot for measuring in subsequent intervals. The concentration of *moringa* within each sample was analyzed using a spectrophotometer (Shimadzu, Kyoto, Japan) at a specific wavelength (274 nm) that was determined by scanning a pure extract of *moringa.* The PBS was used as a blank during measuring.

The concentration was calculated using a specific equation:

Y = mX + b(7)

Y is the absorbance, the slope of the line (m) = 0.0044, X is the concentration of the released *moringa*, and b is the intercept = 0.072.

(8) $$x=y-\frac{0.072}{0.0044}$$

A standard curve for absorbance vs. concentration of moringa was calculated by the Microsoft Excel program.

The cumulative release of moringa was calculated as the ratio of the released amount at time t (M_t_) to the total amount released at equilibrium (M_∞_). To describe the release kinetics, experimental data were fitted to standard models:

- First-order:

$${\frac{M_{t}}{M\infty }=1-e}^{-k1t}$$

- Higuchi:

$${\frac{M_{t}}{M\infty }=K_{H}t}^{1/2}$$

where k_1_ is the first-order rate constant, K_H_ is the Higuchi constant, and n is the release exponent indicating the mechanism. Nonlinear regression was carried out using GraphPad Prism (v 10).

### 2.18. Statistical Analysis

All experiments were performed in triplicate (n = 3) unless otherwise stated, and results are expressed as mean ± standard deviation (SD). Data normality was evaluated using the Shapiro–Wilk test.

Antioxidant, drying time, cytotoxicity, and film thickness assays were analyzed using one-way ANOVA followed by Tukey’s post hoc test.

Antibacterial assay data, which did not meet normality, were analyzed using the Mann–Whitney U test.

Degradation rate, swelling index, and in vitro drug release data were analyzed using two-way ANOVA followed by Bonferroni’s post hoc test. Statistical significance was considered at *p* < 0.05. All statistical analyses were conducted using IBM SPSS Statistics version 29 (IBM Corp., Armonk, NY, USA). Reproducibility was ensured by repeating experiments on independent occasions with freshly prepared samples.

## 3. Results

### 3.1. HPLC

Different phenolic acids and flavonoids were detected within moringa leaf extract as listed in Table 1, with gallic acid exhibiting the highest concentration while quercetin has the lowest. However, chlorogenic acid and myricetin were not detected.

### 3.2. pH

The measured pH for the various formulas is shown in Table 2. PVA showed a slightly acidic pH (6.4). The addition of *moringa* decreased the pH to 5.8.

The hydrogel composite (PVA + SA + HA) showed neutral pH. This buffering capacity is also apparent with a 0.5% *moringa* concentration. However, higher *moringa* concentrations (5–10% w/w) decreased the pH to 5–5.3, respectively.

### 3.3. Antibacterial Assay (Well Diffusion Method)

Only higher concentrations of moringa-containing hydrogels (10% and 20%) showed inhibition zones with no statistical difference between them (see Table 2).

### 3.4. Antioxidant Assay (DPPH Test)

The values of antioxidants’ scavenging activity are presented in Table 2. The hydrogel composite without and with *moringa* showed an antioxidant effect and that the increase in the concentration of *moringa* enhanced the antioxidant properties.

### 3.5. Cytotoxicity Test

**Figure 1:**
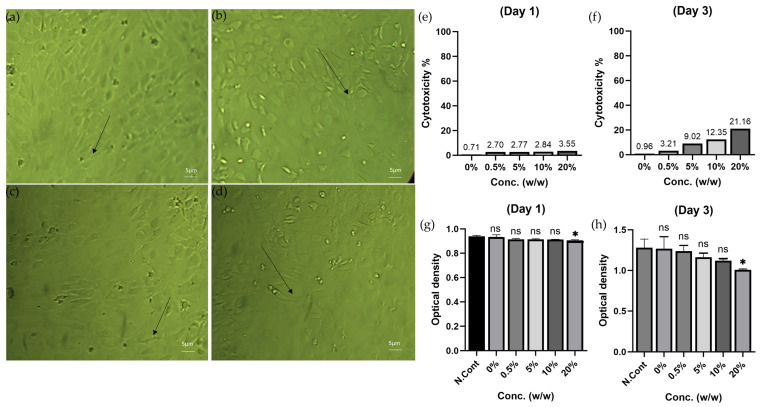
Morphological features (30×) with inhibition rate and optical density of normal human fibroblast cells (**a**): treated cells after one day; (**b**): treated cells after three days; (**c**): untreated cells after one day; (**d**): untreated cells after three days; (**e**): inhibition rate after one day; (**f**): inhibition rate after three days; (**g**): optical density reading curve after one day; (**h**): optical density reading curve after three days. N. Cont. = negative control; Conc. = concentration; ns = non-significant; * = significant difference (*p* ≤ 0.05).

The findings of this study showed minimal or negligible cytotoxic effects of different formulas on the normal human fibroblast cells for two intervals (day one and day three). The arrows mark the normal spindle shape of normal human fibroblasts at all time intervals, as none of the formulas showed cytotoxic features, including cell shrinkage, membrane blebbing, or formation of apoptotic bodies. The micrographs indicate the absence of any sign of cytotoxic effects due to the high density of treated cells, which resemble the control untreated cells, as shown in Figure 1a–d.

Quantitatively, the inhibition rate ranged between 0.71% (hydrogel composite) and 3.55% (20% moringa) at day one, and between 0.96% (hydrogel composite) and 21.16% (20% moringa) at day three (Figure 1e,f). All formulations maintained cell viability well above the ISO 10993-5 threshold of 70%. Optical density readings (Figure 1g,h) further support these findings, with no significant differences compared to the control group for the hydrogel composite, 0.5%, 5%, and 10% moringa formulations. Only the 20% moringa formulation showed statistically lower viability at both 24 h and 72 h.

### 3.6. Drying Time

The hydrogel composite showed a statistically higher drying time than other formulas containing *moringa*. The increase in the concentration of *moringa* in hydrogels from 0.5% to 10% shortened the drying time significantly, as shown in Table 2.

### 3.7. Film Thickness

The film thickness and retraction ratio of different formulas were shown in Table 2. It is apparent that the incorporation of a low concentration of *moringa* did not affect the thickness and retraction ratio. In contrast, the hydrogel composite with high concentration (10% *moringa*) showed higher thickness and lower retraction ratio.

### 3.8. FTIR

**Figure 2:**
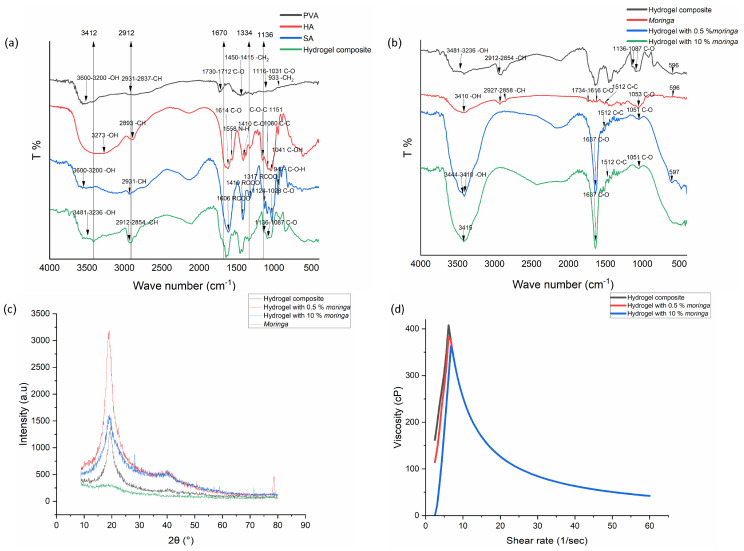
Patterns of different formulas of hydrogels: (**a**) FTIR of elements and hydrogel composite; (**b**) FTIR of hydrogel composite without and with *moringa*; (**c**) XRD; (**d**) shear viscosity.

FTIR in Figure 2a,b shows the chemical functional groups of each hydrogel (PVA, HA, and SA) and chemical interaction between these components in the composite hydrogel without and with *moringa* (0.5% and 10%). For PVA hydrogel, the peaks in the region of 3200–3600 cm^−1^ indicated stretching vibrations of the O–H group. The peak at 1712–1730 cm^−1^ (C=O group) is related to the residual acetate group produced during the hydrolysis of polyvinyl acetate (manufacturing process), which depends on the degree of hydrolysis of PVA.

Regarding HA, the peak at 3273 cm^−1^ is related to the stretching vibration of the O–H and N–H groups. The peaks in the regions of 1410 and 1614 cm^−1^ are related to the stretching of the C=O functional group in ester and amide I bonds, respectively. The spectrum of sodium alginate showed different peaks; the peaks in the region 3200–3600 cm^−1^ are related to the stretching vibration of the O–H group. Peaks at 1028–1124 cm^−1^ are related to the stretching vibration of the C–O functional group of the pyranose ring.

The composite containing a mixture of hydrogels (PVA, HA, SA) showed peaks in the region 3236–3481 cm^−1^, which indicates stretching vibration of the O–H group, also shifting of the peak in wave number, and changes in the depth and width of the band. When pure sodium alginate was compared with a composite of hydrogels, the addition of polyvinyl alcohol and hyaluronic acid to sodium alginate led to shifting of peaks in the region of C=O and C–O to higher wave numbers

The ethanolic extract showed different peaks; the peak at 3410 cm^−1^ is related to the stretching vibration of the O-H group, which belongs to polyphenols. The bands in the 1616–1734 cm^−1^ region are related to the functional group C=O and come from polar compounds. For hydrogels containing *moringa*, peaks are detected in the regions (3410 cm^−1^, 3415 cm^−1^, and 3444 cm^−1^) that indicate the stretching vibration of O-H groups; the addition of *moringa* increased the width and intensity of the bands, and the increase in concentration from 0.5% to 10% led to the disappearance of the peak that was originally found in 3444 cm^−1^ and shifting in the wave number from 3410 cm^−1^ to 3415 cm^−1^. The region of the C=O bond exhibited a new sharp peak at 1637 cm^−1^. The C–O functional group showed shifting to the lower wave number 1051 cm^−1^ in comparison with pure *Moringa oleifera* extract 1053 cm^−1^.

### 3.9. XRD

The pattern of XRD indicated that the hydrogel composite has a distinct peak at 2θ = 19.5° and a less pronounced peak at 2θ = 40°.

The incorporation of *moringa* at low concentrations (0.5%) resulted in an increase in the intensity of peaks at 2θ = 19.5° and 2θ = 40°. Furthermore, a new peak emerged at 2θ = 78.5°. The higher concentration of *moringa-contained* hydrogels showed a prominent peak only at 2θ = 19.5° with the disappearance of the peak at 2θ = 40°, as shown in Figure 2c.

### 3.10. Shear Viscosity

The shear viscosity showed two stages of behavior (see Figure 2d). The first stage includes shear thickening behavior. In the second stage, all formulas showed shear thinning behavior, and viscosities of all formulas became similar in relation to shear rate.

The formulas of hydrogel composite, with 0.5%, and with 10% *moringa*, showed the highest viscosity: 407 CP, 383 CP, and 363 CP, respectively.

### 3.11. SEM

**Figure 3:**
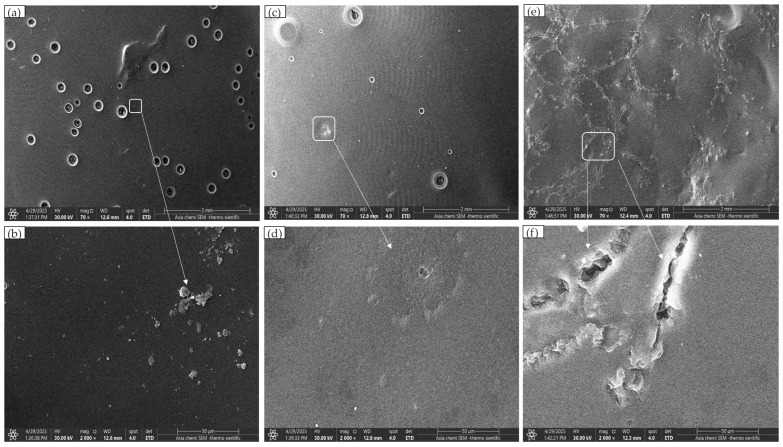
SEM images of three formulas: (**a**) hydrogel composite overview (70×); (**b**) hydrogel composite magnified view (2000×); (**c**) hydrogel with 0.5% *moringa* overview (70×); (**d**) hydrogel with 0.5% *moringa* magnified view (2000×); (**e**) hydrogel with 10% *moringa* overview (70×); (**f**) hydrogel with 10% *moringa* magnified view (2000×).

The hydrogel composite formula showed a homogenous film structure with multiple pores (Figure 3a); additionally, the higher magnification clarifies bright, scattered aggregates that are related to PVA crystals, as marked by the arrow in Figure 3b. With the incorporation of a low concentration of moringa into the hydrogel film of the 2nd formula, the film remained homogenous with a reduction in pore sizes and number; the moringa acted as a filler, blocking some pores, as shown in the overview and magnified images (Figure 3c,d). The 3rd formula, which has a high concentration of moringa (10%), showed phase separation and aggregation of moringa at a specific area; no available pores were detected. Instead, there are multiple defects crossing the matrix, as marked by arrows (Figure 3e,f).

### 3.12. Folding Endurance

Hydrogel composites and hydrogels containing a low concentration of *moringa* (0.5%) passed 300 folds without fracture. With the increase in the concentration of *moringa* (10%), the film broke after 12 cycles.

### 3.13. Degradation Rate

**Figure 4:**
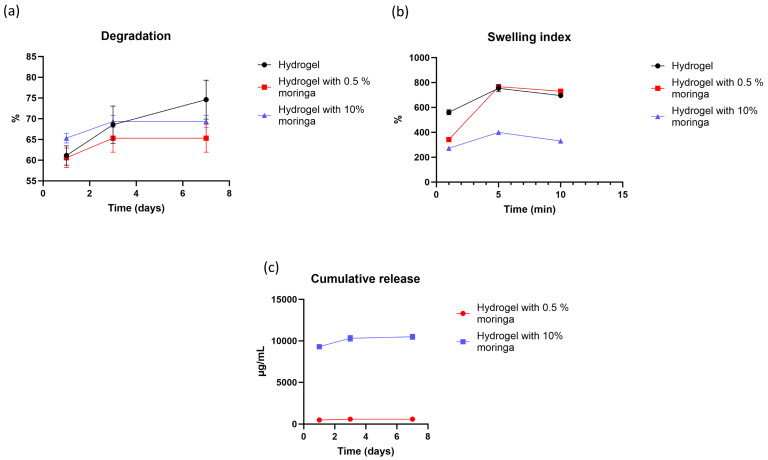
Illustration of different formulas of hydrogels: (**a**) degradation rate; (**b**) swelling index; and (**c**) *moringa* release.

The values of degradation rate for all formulas at different time intervals are shown in Figure 4a. The time factor statistically affects the degradation of films; the degradation rate statistically increased with time (d1, d3, and d7) (*p* < 0.001). In contrast, the formula did not statistically increase or decrease the solubility of films (*p* = 0.113). The interaction between time and formula factors is significant (*p* = 0.016), which is clarified by the effect of formulas containing 0.5% and 10% of moringa on the degradation of the second and third intervals, as the degradation of films containing moringa only increased during the two intervals (d1 and d3) and remained stable on the third interval (d7).

### 3.14. Swelling Index

The values of the swelling index that represent all formulas are presented in Figure 4b. For all formulas (hydrogel composite, with 0.5%, and with 10% *moringa*), the swelling index increased significantly from the first to second interval, reaching the maximum value (equilibrium), and then the swelling index decreased significantly (the time factor statistically affected the swelling index, *p* < 0.001). The formula factor statistically affects the swelling index (*p* < 0.001); the incorporation and increasing the concentration of *moringa* in the 2nd and 3rd formulas decreased the swelling index significantly. In addition, the time–formula factor is statistically significant (*p* < 0.001).

### 3.15. In Vitro Drug Release

For two formulas (hydrogel with 0.5% and hydrogel with 10% *moringa*), the highest released concentration of *moringa* was found in the day one interval for both concentrations (burst release); in the 2nd and 3rd intervals, they showed a lower release of *moringa* with a sustained pattern (time statistically affected the release, *p* < 0.001). The effect of the formula is statistically apparent; the formula with higher concentration (10% *moringa*) has higher release than the lower concentration (0.5% *moringa*) (*p* < 0.001); additionally, the time-formula statistically affected the release of *moringa* (*p* < 0.001). The cumulative release of *moringa* for three periods of two concentrations is presented in Figure 4c.

The cumulative release profiles showed that moringa was rapidly released, with >80% detected within the first day for both formulations. Nonlinear regression indicated that the first-order model best described the release data (0.5% moringa: R^2^ = 0.9997, K_1_ = 1.73° day^−1^; 10% moringa: R^2^ = 0.9997 K_1_ = 2.17° day^−1^). The Higuchi model also fitted the data reasonably well (0.5% moringa: R^2^ = 0.68, K_H_ = 0.47 day^−1^; 10% moringa: R^2^ = 0.62 K_H_ = 0.48 day^−1^), consistent with diffusion-controlled release.

## 4. Discussion

The primary objective of this study is to characterize and develop pulp-capping material. With specific properties to overcome drawbacks associated with conventional materials, this pulp-capping material could produce a favorable outcome of dentin–pulp complex repair. The two formulas containing hydrogel composites without and with 0.5% *moringa* could show excellent biocompatibility, bioactivity, inflammatory exudate absorption, degrade in a specific time, and could guide the tissue toward healing and repair. The material should have satisfactory applicability and set within a reasonable time for clinical success; therefore, the use of hydrogel composites (PVA, HA, SA) with well-known bioactive *moringa* was formulated for this purpose.

PVA is an inexpensive, tasteless, odorless, biocompatible synthetic polymer with high water retention and excellent mechanical properties [22,23]. However, this hydrogel lacks flexibility and hydrophilicity [16]. The addition of other natural source hydrogels such as HA and SA could enhance water retention, inflammatory exudate absorption, immunomodulatory, and antioxidant scavenging effects [24,25,26,27,28]. pH could play crucial role in the selection of the composite over PVA hydrogel alone; the hydrogel composite of PVA, HA, and SA showed a neutral pH due to the buffering capacity of sodium alginate, while PVA hydrogel alone showed an acidic pH resulting from residual acetyl groups, as confirmed by the pH test [29].

Several concentrations of PVA, HA, and SA were tried until they reached the selected concentration and proportions: 60% of 10% PVA, 20% of 0.25% HA, and 20% of 3.75% SA, respectively. The selection was determined by the handling properties during preparation and suitable viscosity during application. The increase in PVA concentration beyond 10% caused agglomeration of the mixture even at temperatures higher than 90 °C; additionally, the use of high molecular weight HA is mandatory due to its anti-inflammatory and water retention properties, this could restrict the use of higher concentrations of hydrogel in the mixture, allowing ideal handling properties.

*Moringa oleifera* ethanolic extract was selected for this study according to the result of a previous study performed by Shafiq and Mahdee; ethanolic extract has an immunomodulatory effect, antibacterial (*S. mutans* and *E. faecalis*) and antifungal properties (*C. albicans*), comparable cytotoxicity with MTA (both of them within ISO standards), upregulation of alkaline phosphatase enzyme, and formation of calcified nodules better than MTA [10]. Unlike conventional cross linkers such as glutaraldehyde and borax with known toxicity, *Moringa oleifera* extract has natural cross-linker compounds such as quercetin and tannins. These cross-linkers are biocompatible, antibacterial, antioxidant, anti-inflammatory, polymer binders, and modifiers of mechanical and physical properties of hydrogels. These polyphenols can form hydrogen bonding (physical interaction) with polymer functional groups [30,31,32,33].

The hydrogel composite without *moringa* showed a successful homogenous structure with multiple pores confirmed by SEM; this porosity is related to the presence of the amorphous structure of SA in the hydrogel composite, and this porosity is essential for carrier swelling, allowing inflammatory exudate absorption [34,35]. The hydrogel composite had higher shear thickening viscosity for clinical application than other formulas, which is related to the reformation of hydrogen bonds between hydroxyl groups and the jamming of molecules bound by hydrodynamic force at a low shear rate [36]. The mechanical property of this composite was reasonable, passing 300 folds without fracture [37].

The hydrogel composite (PVA, HA, and SA) exhibited antioxidant properties, despite the absence of phenolic compounds, potentially due to the antioxidant effect of hyaluronic acid and sodium alginate [27,28]. Regarding the biocompatibility of hydrogel composite extract, this formula showed excellent biocompatibility and no difference when compared with the control group, which is related to the biocompatibility of each element [35,36]. Each element is known for its role in wound healing and tissue engineering, allowing cell migration, signaling, and attachment [38,39].

Several concentrations of *moringa* (0.5–20%) were tried and included in hydrogel formulas. Only two concentrations (0.5% and 10%) were selected for further testing and compared with hydrogel composites; the selection was performed according to the results of pH, antibacterial, antioxidant, and cytotoxicity. The compromise between antibacterial effects and cytotoxicity is crucial; therefore, the low concentration (0.5%) *moringa*-containing hydrogel with antioxidants, neutral pH, and high biocompatibility was selected.

Another formula containing a high concentration of *moringa* (10%) with antibacterial, antioxidant, acidic pH, and lower biocompatibility was selected. The preference of the formula with 10% *moringa* over 20% is related to the lower biocompatibility, lower pH, and limited enhancement of antibacterial action (statistically non-significant difference) of the latter.

Additionally, the decrease in pH is associated with decreasing ionization of carboxyl groups (functional groups of HA and SA), decreasing electrostatic repulsion of functional groups, and reducing the swelling property of hydrogels; consequently, it restricts the release of bioactive *moringa* [40,41].

With the incorporation of *moringa* at a low concentration (0.5%), the hydrogel surface became denser, with fewer visible openings, suggesting reduced surface porosity (confirmed by SEM); the reduction in the number of pores in comparison with the hydrogel composite without *moringa* could be related to the filler behavior of *moringa* [42,43]. In contrast, the higher concentration of 10% *moringa* blocked the pores completely with phase separation and surface cracking. These pores are essential for the swelling properties, as they allow for exudate absorption associated with injury, reduce intrapulpal hyperaemia, retain moisture for cell viability, and enhance the healing process [34,35]. The same property is essential when using hydrogels as a wound dressing [44]. These observations are consistent with the swelling and solubility results, which also indicated reduced water uptake and lower solubility at higher moringa concentration. Although our current analysis is limited to surface SEM, future studies with cross-sectional SEM would provide more direct insights into internal pore networks.

The physical or chemical interaction of *moringa* with hydrogel composites was shown in the FTIR test, as the presence of shifting in the wave number and the appearance of new peaks could indicate the occurrence of such an interaction [45,46]. The observed FTIR shifts in the C=O and C–O stretching regions upon incorporation of moringa extract indicate intermolecular interactions between the hydrogel polymers and the polyphenols of moringa. The phenolic and hydroxyl-rich constituents of moringa are capable of forming hydrogen bonds with the carbonyl and hydroxyl groups of PVA, SA, and HA [47]. Additionally, the XRD test confirms the presence of this interaction, as there was an increase in the intensity of the peak at 2θ = 19.5° and the appearance of new peaks at 2θ = 40° and 78.5°; these peaks indicate an increase in the crystallinity of hydrogels (especially PVA) [48]. Despite the amorphous nature of *moringa* extract, polymer–extract interactions explain the increase in crystallinity in the hydrogel upon its incorporation. The presence of *moringa* could rearrange the PVA chains, promote tighter chain alignment, and increase the crystallinity of the PVA structure [48]. Therefore, the increase in crystallinity is related to the change in the polymer itself not from *Moringa oleifera* extract.

The addition of *moringa* at low concentrations is associated with increasing the antioxidant scavenging effect, which is related to phenolic acid and flavonoids (confirmed by HPLC). These anti-inflammatory and antioxidant characteristics facilitate the reduction in tissue damage and promote tissue proliferation and regeneration [49]. This low concentration of *moringa* (0.5%) does not jeopardize the mechanical properties as detected in the folding endurance test; the hydrogel film with low concentration (0.5%) passed 300 cycles without fracture.

The scenario is different when a high concentration of *moringa* is added to a hydrogel. There is early failure in the folding endurance test, a decrease in the crystallinity (XRD), multiple defects (SEM), a lower swelling index, lower viscosity, and high solubility. This deterioration is related to the plasticizer effect of *moringa* that prevents hydrogen bonding between hydroxyl groups of hydrogels and the high hydrophilicity of this extract [50,51,52,53,54]. The formula factor (without and with different concentrations of *moringa)* did not affect the degradation rate of the films; this could be related to the low concentration of the cross-linker (quercetin) in the whole crude extract [31,55].

The drying time is shortened by addition and increasing *moringa* concentration; the presence of quercetin acts as a cross-linker for the polyvinyl alcohol structure, as this compound has methoxy and hydroxyl groups that react with the hydroxyl group of polyvinyl alcohol [31]. At the highest concentration of *moringa*, the shortening of drying time could be related to the increasing of solid hydrophobic components and the formation of a supersaturated solution, and this in turn will lead to faster loss of the water from the matrix [56].

The shear viscosity has two-stage behavior. The first stage includes shear thickening behavior; this is related to the reformation of hydrogen bonds between hydroxyl groups and jamming of molecules bound by hydrodynamic force at low shear rate [36]. In the second stage, the shear thinning behavior at higher shear rates is related to increasing the distance between molecules and the disintegration of hydrogel by disruption of hydrogen bonds and chain entanglement [36].

The incorporation of 10% w/w of *moringa* resulted in an increase in the film thickness. The increase in concentration led to the formation of a supersaturated solution and the formation of a precipitate. This precipitate acts as a plasticizer, which decreases hydrogen bonding between hydroxyl groups of PVA. The hydrogen bonding between these groups is crucial for polymerization and film formation; consequently, *moringa* acting as a plasticizer mitigates the retraction ratio (polymerization shrinkage) during film formation [52,53]. This result corroborates the findings of Gheorghita et al., as they observed an increase in film thickness with the addition of *moringa* powder [57].

For two formulas (hydrogel with 0.5% and hydrogel with 10% *moringa*), the highest released concentration of *moringa* was found in the day one interval; this burst release may be related to the high solubility of highly hydrophilic and un-crosslinked parts of hydrogels and accumulated *moringa* on the surface of the hydrogel that passes from the bulk of the film to the surface during the drying process [58,59,60]. The sustained release pattern of *moringa* is related to swelling properties that allow the gradual passage of molecules through hydrogel pores. In addition, the remaining physically cross-linked part of hydrogels may explain the gradual loss of *moringa* from the polymer [31,61,62]. All three intervals (d1, d3, and d7) of hydrogels with 10% of *moringa* were statistically higher than the intervals of another concentration (0.5%). This difference can be attributed to the higher drug loading, which confirms that the formulas exhibited concentration-dependent diffusion; the higher concentration resulted in greater *moringa* release following first-order kinetics [63]. Fitting the data to the Higuchi model also produced a constant of approximately K_H_ = 0.47 for both concentrations, consistent with diffusion-controlled release. The similarity of values reflects the rapid burst release (>80% within the first day), which limited the ability of the Higuchi model to discriminate between groups. Furthermore, due to the absence of the early stage region (Mt/M∞ ≤ 0.6), the Korsmeyer–Peppas model could not be applied. Nevertheless, the first-order analysis clearly differentiated between concentrations, with K_1_ = 1.73 day^−1^ for the 0.5% formulation and K_1_ = 2.17 day^−1^ for the 10% formulation, supporting the concentration-dependent diffusion mechanism [43].

Antibacterial action was only established at high concentrations of *moringa-loaded* hydrogels (10% and 20%), as high concentrations of phenolic compounds (flavonoids and phenolic acids) are required to disrupt bacterial cell walls, inhibit bacterial mobility, and avoid virulence factors [64]. The result of this study is in agreement with the literature, as they found minimal antibacterial activity of plant extract on different bacterial species and fungi [8,65]. The high solubility and burst release of *moringa* are related to un-crosslinked formulas. The selection of hydrogels without cross-linkers could be justified by two points: the first is that avoiding cross-linkers is mandatory to achieve appropriate viscosity with adequate clinical spreadability; additionally, the pulpal fluid is limited, and using the cross-linker may avoid the adequate release of the bioactive compounds of *moringa.*

The main limitation of this study is the absence of a histological evaluation to estimate its biological effect regarding pulp-dentine complex repair, as this study is an in vitro study; additionally, the tests (swelling index, degradation rate, and *moringa* release) were performed in conditions that do not mimic the biological system, including flow rate, fluid volume, and presence of an enzymatic system, which may affect degradation.

## 5. Conclusions

The hydrogel formula exhibited desirable physical, chemical, and biological properties; the incorporation of *moringa* at low concentrations enhanced structural integrity and scavenging activity without compromising cell viability. Increasing the concentration of bioactive *moringa* to the highest levels compromises the mechanical properties in spite of the enhancement of the antibacterial and antioxidant properties. Therefore, the use of hydrogel composites (PVA, HA, and SA) with a low concentration of *moringa* (0.5%) may be considered a promising candidate as a novel pulp-capping agent supporting tissue healing and regeneration.

## Figures and Tables

**Table 1:** HPLC values of calculated concentrations of flavonoids and phenolic acids.

Phenolic Compound	Area (Standards)	Area (Sample)	Concentration (Sample) µg/mL
Gallic acid	45,793	320,026	69.88535
Vanillic acid	175,796	179,648	10.21912
Quercetin Anhydrous	59,797	33,161	5.545596
Apigenin	20,523	16,078	7.834137
Kaempferol	94,304	73,597	7.804229
Chlorogenic acid	10,927	-	-
Myricetin	2385	-	-
Rutin	29,912	27,766	9.282562
Isorhamnetin	9484	8585	9.052088

**Table 2:** Characterization values of different physical and biological tests.

Name of Material	Concentration of *moringa*	pH	Antibacterial Assay, Inhibition Zones (mm) (±SD)	Antioxidant Assay, Inhibition Rate (%) (±SD)	Drying Time (s) (±SD)	Film Thickness (±SD) and [RR %]
PVA	-	6.4	-	-	-	-
PVA + moringa	0.5% W/W	5.8	-	-	-	-
PVA + HA + SA	-	7.3	No inhibition	23.83% ^A^ (0.036)	218.33 ^A^ (7.63)	0.054 ^A^ (0.006) [96.72%]
Hydrogel + moringa	0.5% W/W	7.2	No inhibition	37.7% ^B^ (0.264)	146.33 ^B^ (5.5)	0.054 ^A^ (0.004) [96.72%]
	5% W/W	5.3	No inhibition	39.8% ^C^ (0.36)	-	-
	7.5% W/W	5.1	No inhibition	40.1% ^C,D,E^ (0.1)	-	-
	10% W/W	5	1.03 ^A^ (0.057)	40.4% ^C,D,E^ (0.2)	45 ^C^ (5)	0.115 ^B^ (0.004) [93.06%]
	20% W/W	4.8	1.33 ^A^ (0.057)	40.6% ^E^ (0.2)	-	-

The same superscript capital letter in the same column indicates no statistical difference (*p* > 0.05), while different superscript capital letters in the same column indicate statistical difference (*p* ≤ 0.05).

## Data Availability

The original contributions presented in this study are included in the article. Further inquiries can be directed to the corresponding author(s).

## Abbreviations

The following abbreviations are used in this manuscript:

HPLC	High performance liquid chromatography
PVA	Polyvinyl alcohol
HA	Hyaluronic acid
SA	Sodium alginate
SEM	Scanning electron microscope
FTIR	Fourier transform infrared spectroscopy
XRD	X-ray diffraction
DPPH	2,2-diphenyl-1-picrylhydrazyl
NHF	Normal human fibroblast
MEM	Minimum essential media
FBS	Fetal bovine serum
DMSO	Dimethyl sulfoxide
PBS	Phosphate buffer saline
